# Primary vaginal calculus in a woman with urogenital sinus anomaly: a case report

**DOI:** 10.1186/s12894-020-00708-0

**Published:** 2020-09-04

**Authors:** Qiong Xu, Yu Zou

**Affiliations:** Department of Radiology, Women’s Hospital School of Medicine Zhejiang University, Hangzhou, 310006 Zhejiang province China

**Keywords:** Urogenital sinus, Vaginal calculus, Computed tomography, Magnetic resonance imaging

## Abstract

**Background:**

Primary vaginal calculus is rare and often misdiagnosed due to its low incidence. The formation of primary vaginal calculus is mainly due to the pooling and stasis of urine within the vagina, and associated with urogenital tract abnormalities.

**Case presentation:**

We present a case of a 23-year-old woman with urogenital sinus anomaly who presented with a vaginal calculus. The patient was not suspected of a calculus in the vagina until the patient suffered amenorrhea and dyspareunia. Pelvic computed tomography (CT) and magnetic resonance imaging (MRI) confirmed the patient had urogenital sinus anomaly with vaginal calculus. For the reason, the calculus was removed by surgery, and the reconstruction of vagina and urethra was performed. The postoperative recovery and follow-up were uneventful.

**Conclusions:**

Although vaginal calculus and urogenital sinus anomaly are extremely rare in literature, the radiologist should be familiar with the imaging appearance of urogenital sinus anomaly, and be aware of the possibility of vaginal calculus.

## Background

Primary vaginal calculi are rare disorder which has been reported in association with urogenital tract abnormalities such as urogenital sinus anomaly, urethrovaginal fistulas, vaginal outlet obstruction and bladder exstrophy [1–5]. This report describes of a primary vaginal calculus caused by urogenital sinus anomaly.

## Case presentation

A 23-year-old woman was referred to our hospital because of recurrent abdominal pain for 7 years, amenorrhea and dyspareunia. The patient’s mental state and movement ability was normal. She had a history of fracture of both legs and skin injury of pubic caruncle caused by trauma about 20 years ago.

Gynecological examination showed the patient had a single orifice on the urogenital region located where the urethra would be expected. There is a normally located anal perineal orifice. When forced urination, urine flowed out of the single orifice on the urogenital region. The urethral catheter was inserted about 70 mm through the single orifice, and no urine flowed out.

**Fig. 1:**
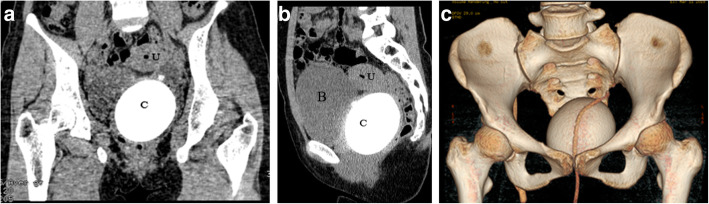
The multiple planar reconstruction (MPR) (**a**, **b**) and volume rendering (VR) (**c**) of pelvic CT, showing a large calculus in the vagina. B, bladder; U, uterus; C, calculus

**Fig. 2:**
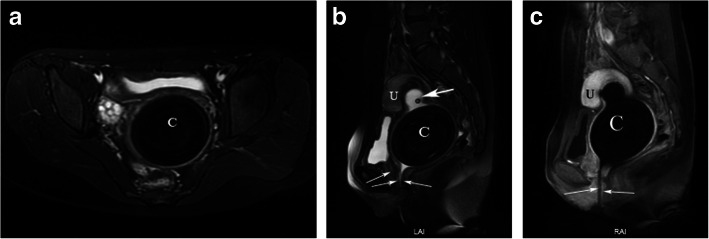
**a** Axial and **b** Sagittal T2-weighted imaging with fat saturation of pelvis, showing a large, extremely low-signal intensity mass (**c**) in the vagina that caused the uterus moved upward. A balloon catheter shadow (thick arrow) can be seen above the mass. The urethra and vagina form a common channel (arrow). **c** Sagittal contrast-enhancement T1-weighted imaging with fat saturation of pelvis, showing the mass has no enhancement

Pelvic CT scan showed a large calculus and a urethral catheter in the vagina (Fig. 1a, b, c). Pelvic MRI scan demonstrated the urethra and the vagina merged into a common channel about 3.45 cm long (Fig. 2b, c), and a large calculus about 7.8 cm × 6.8 cm × 7.7 cm in size lodging in the upper vagina, the uterus was moved upward (Fig. 2a,b,c); a urethral catheter entered the vagina through the opening on the urogenital region; both ovaries and bladder were normal and no other congenital malformation was noted (Fig. 2a). Transabdominal sonography imaging revealed a vaginal stone, and normal two kidneys, the bladder, uterus, cervix, and adnexa.

**Fig. 3:**
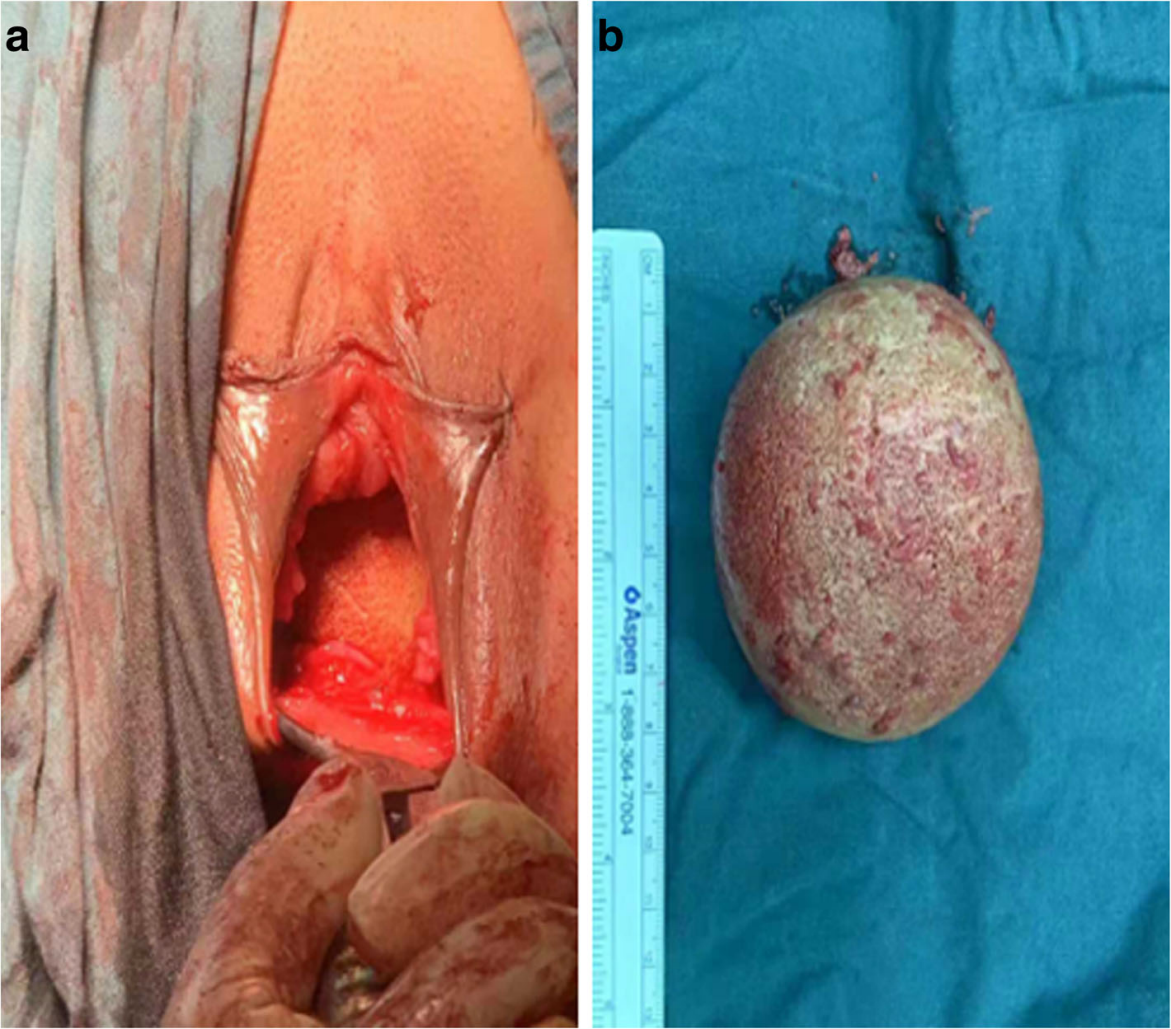
**a** Vaginal calculus is seen through the median perineum incision in the vagina during its removal; **b** Image of the vaginal calculus removed during surgery

Under general anesthesia, entering the urogenital tract through the median perineum approach and a 2-cm incision was performed (Fig. 3a). A hard grayish-yellow abnormal mass was observed in the vaginal cavity (Fig. 3b), and there was no adhesion to the surrounding mucosa. The tissue around the calculus was carefully separated and the calculus was completely removed.

During the exploration after lithotomy, the common channel formed by vagina and urethra was detected, and the orifice of urethra was about 3 cm from the anterior vault of the vagina. The urethral catheter was inserted about 20 mm through the orifice of urethra, the urine flow was observed. Therefore, the estimated length of the urethra was about 2 cm. Hysteroscopic insertion of the vagina revealed thin endometrium and normal cervix, no other abnormality was found. The patient was diagnosed with a vaginal stone, urogenital sinus anomaly and thin endometrium.

Vaginal reconstruction (vaginoplasty) and urethra reconstruction (urethroplasty) were performed. The mucosa near the urethral orifice was taken and wrapped by 20 fr Foley’s catheter, forming a urethra about 4 cm and neourethral meatus. The vaginal mucosa was pulled down and intermittently sutured with the perineal skin to form a vagina about 8 cm that could hold two fingers.

After the operation, the patient received the treatment of estrogen and progesterone, and menarche appeared 20 days later. One month after the operation, the patient recovered well, and could urinate by himself. After 8 months of follow-up, the patient had normal menstruation and urination. The patient is married and ready to conceive.

## Discussion and conclusion

Vaginal calculi are extremely rare and are classified as primary and secondary. The cause of primary vaginal calculi is probably caused by the pooling and stasis of urine within the vagina. In the literature, primary vaginal calculi are more frequently associated with urogenital tract abnormalities [1–5]. In our case, the urogenital sinus was undoubted the underlying cause of calculi formation. Urine flowed back into the vagina from the common channel, and was prone to stagnation, which seemed to help the formation of calculi.

In most cases, the clinical manifestations of patients are not specific. Most of the symptoms are caused by enlarged stones in the vagina, including infections, vaginal pain, dyspareunia, urinary incontinency [2, 5–8]. In our study, the patient presented with recurrent abdominal pain, amenorrhea and dyspareunia. At the beginning of the treatment, the patient was not considered to have stone in the vagina until US, CT and MRI examinations.

B ultrasound and X-ray examinations of the pelvis are helpful to confirm the diagnosis of calculi, but it is difficult to determine the specific location of calculi by X-ray examination, and difficult to determine the relationship between large calculi and the pelvic organs by B ultrasound. Pelvic CT can show the exact position, structure and density of the calculi. Pelvic MRI can clearly show the location of the calculi, the structure of vagina and urethra as well as the endometrium and muscle layers. Therefore, CT and MRI examinations have strong advantages in distinguishing pelvic stones and correctly diagnosing vagina stones. In our study, multiple examinations such as gynecologic examination, CT, and MRI examinations can be used to accurately diagnose urogenital sinus with vaginal calculus.

The patient was amenorrhea, and hysteroscopy found that the patient’s endometrium was thin. There are many reasons for the formation of thin endometrium and amenorrhea, including age, drugs, mechanical damage and so on. In our study, we believe that thin endometrium was the main cause of amenorrhea. Urine retained in the vagina was easy to enter the uterine cavity, stimulating and inhibiting endometrial hyperplasia. After the operation, the menstruation of the patient recovered after the treatment of estrogen and progesterone.

To our knowledge, only one case has been previous reported of vaginal calculus associated with an isolated urogenital sinus anomaly [1]. In the case, the patient was a 3-year-old girl who successfully completed endoscopic intracorporeal lithotripsy, and reconstructive surgery was postponed until puberty. In our study, the patient underwent open surgery to remove the calculus completely and reconstruct the vagina and urethra. The prognosis was good.

## Data Availability

Not Applicable.

## Abbreviations

CT: Computed tomography
MRI: Magnetic resonance imaging
MPR: Multiple planar reconstruction
VR: Volume rendering
US: ultrasonic
